# IMPROV-ED trial: eHealth programme for faster recovery and reduced healthcare utilisation after CABG

**DOI:** 10.1007/s12471-020-01508-9

**Published:** 2020-11-03

**Authors:** G. J. van Steenbergen, D. van Veghel, J. ter Woorst, D. van Lieshout, L. Dekker

**Affiliations:** 1 Catharina Heart Center, Catharina Hospital, Eindhoven, The Netherlands; 2 Dutch Heart Foundation, The Hague, The Netherlands

**Keywords:** eHealth, Video consultation, Patient education, Healthcare utilisation, Emergency department visits, Coronary artery bypass grafting

## Abstract

**Background:**

After coronary artery bypass grafting (CABG), healthcare utilisation is high and is partly unplanned. eHealth applications have been proposed to reduce healthcare consumption and to enable patients to get actively involved in their recovery. This way, healthcare expenses can be reduced and the quality of care can be improved.

**Objectives:**

We aim to evaluate whether the use of an eHealth programme can reduce unplanned healthcare utilisation and improve mental and physical health in the first 6 weeks after discharge in patients who underwent CABG. In addition, patient satisfaction and use of the eHealth programme will be evaluated.

**Methods:**

For this single-centre randomised controlled trial, at least 280 patients referred for CABG will be included at the preoperative outpatient clinic and randomised to an intervention or control group. The intervention group will have access to an eHealth programme, which consists of online educational videos developed by the Dutch Heart Foundation and postoperative video consultations with a physician. The control group will receive standard care and will not have access to the eHealth programme. The primary endpoint is healthcare utilisation; other endpoints include anxiety, duration of recovery, quality of life and patient satisfaction. Participants will complete several questionnaires at 6 time points during the study.

**Results:**

Patient enrolment started in February 2020 and completion of the follow-up period is expected in August 2021.

**Conclusion:**

This randomised trial was initiated to test the hypothesis that patients who are partaking in our eHealth programme use less unplanned care and experience a better quality of life, less anxiety and a faster recovery than controls.

## Introduction

Coronary artery bypass graft grafting (CABG) is the most prevalent cardiac surgery performed in the Netherlands, with roughly 7000 procedures annually [1]. The care chain of CABG is costly, and several quality improvement initiatives have been successfully implemented that sought to contain costs and to improve patient outcomes [2, 3]. Despite the positive effects of these initiatives on costs, mortality, postoperative morbidity and process measures such as in-hospital length of stay, healthcare utilisation in the first 30 days after CABG remains an issue, placing a significant burden on the healthcare system. Readmissions after CABG are commonly reported and the readmission rate can be as high as 34% in the first 30 days [4, 5].

Insight into unplanned healthcare utilisation during this period is scarce (apart from readmissions), but it is reasonable to expect a short hospital stay after CABG is counterbalanced by the use of other healthcare services, especially because planned care is not initiated until 6 weeks after discharge. In this period, patients commonly experience psychological symptoms (e.g. anxiety, depression), have to deal with uncertainty and worry about what to expect (e.g. what level of postoperative pain is normal, is physical exercise allowed?) [6]. Recall of information provided perioperatively is often incomplete and patients do not always know who to contact in case of complaints. They will then search for (sometimes unreliable) information on surgery or recovery and reach out to different healthcare providers, who have a varying degree of expertise in CABG care. Conflicting advice on recovery can further increase fear and insecurity, which will eventually hamper the recovery process and contribute to unplanned healthcare utilisation [7, 8].

We hypothesise that restructuring the postoperative period with an eHealth strategy will reduce unplanned healthcare utilisation through improved mental and physical health and faster recovery. In the IMPROV-ED trial, we aim to evaluate whether the use of an eHealth programme that consists of educational videos developed by the Dutch Heart Foundation (*Hartstichting*) and video consultations, is more effective than standard care in the reduction of unplanned healthcare utilisation and the improvement of patient outcomes in the first 6 weeks after CABG. In addition, a process and patient satisfaction evaluation of the newly developed eHealth strategy will be conducted.

## Methods

### Study setting

This randomised trial is conducted at the Catharina Hospital in the Netherlands. The trial will be reported in accordance with relevant sections from the Standard Protocol Items: Recommendations for Interventional Trials [9], and the Consolidated Standards of Reporting Trials of Electronic and Mobile Health Applications and Online Telehealth. The study was approved by the local medical ethics committee (registration number R19.100) and is registered in the Netherlands Trial Registry (www.trialregister.nl, number NL8510).

### Recruitment and allocation

All patients planned for preoperative outpatient counselling for CABG will be contacted by telephone in the week of their scheduled appointment. Patient eligibility for the trial will be assessed according to prespecified inclusion criteria (Tab. 1). Eligible patients will be informed about the study protocol by one of the investigators after their scheduled appointment. If the patient is willing to participate, the informed consent form is signed. At inclusion, patients are randomised to either the intervention or control group in a ratio 1:1 using block randomisation, with a block size of four.

**Table 1:** Eligibility criteria

Criterium	
1	>18 years of age planned for elective, isolated CABG/OPCAB
2	Sufficient computer knowledge, and internet access. Children can assist, but patients should be able to access their own email and navigate the internet to use the provided eHealth strategy
3	Access to computer with internet connection and webcam or build-in camera
4	Comply to minimal specifications for use of video consultation:
	– PC/laptop: Windows 7 or 10 with Chrome or Firefox browser
	– Android tablet: at least Nougat software installed and use of Chrome browser
	– Apple iPad: at least iOS 12.3.4
5	Ability to speak, read and interpret the Dutch language
6	Provide informed consent

*CABG/OPCAB* coronary artery bypass grafting/off-pump coronary bypass

### Interventions

#### Control group

Patients randomised to the control group will receive standard care according to the local protocol. One month prior to surgery, patients are invited to the preoperative outpatient clinic, where they are individually counselled by a physician and a nurse practitioner. They are also handed information brochures after a nurse-led group session during which they receive information on the CABG care process at our hospital.

After surgery, patients have an in-hospital physiotherapist consultation. Before discharge, a resident or nurse provides brief information about the permitted level of physical activity and the medication schedules and answers remaining questions patients might have. Outpatient postoperative follow-up is scheduled for 6 weeks after discharge. Structured guidance to improve general condition and strength by a physiotherapist is offered (cardiac rehabilitation). Patients have no planned care in the first 6 weeks after discharge.

#### Intervention group

**Fig. 1:**
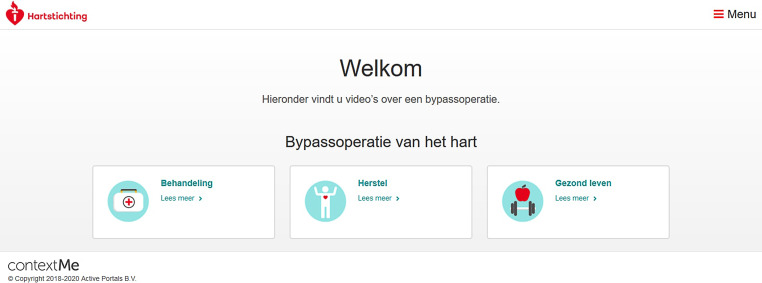
Welcome screen of the eHealth programme with stratification of topics on which patients can find information: treatment (*Behandeling*), recovery (*Herstel*) and healthy living (*Gezond leven*)

**Fig. 2:**
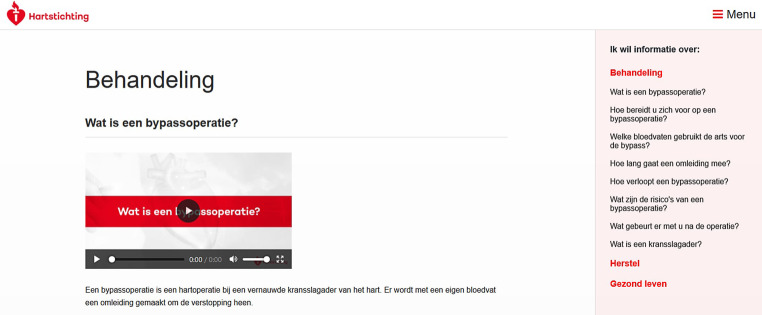
Overview of the portal for the eHealth programme

Aside from standard care as described in the previous section, patients in the intervention group will have access to educational videos and will be invited to two video consultations. Access will be granted through a link sent by email. The educational videos will be made available directly after randomisation for the duration of the study, via a secure online portal. The portal provides an orderly index in which patients can navigate by means of preformulated questions stratified in three categories: treatment, recovery and healthy living (Fig. 1). Each question will be accompanied by an educational video, which provides information and practical advice if applicable (Fig. 2).

**Fig. 3:**
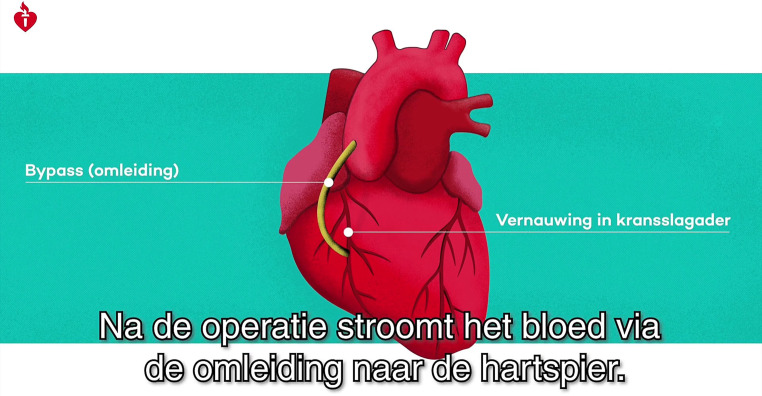
Still from the video ‘What is coronary artery bypass surgery?’

**Fig. 4:**
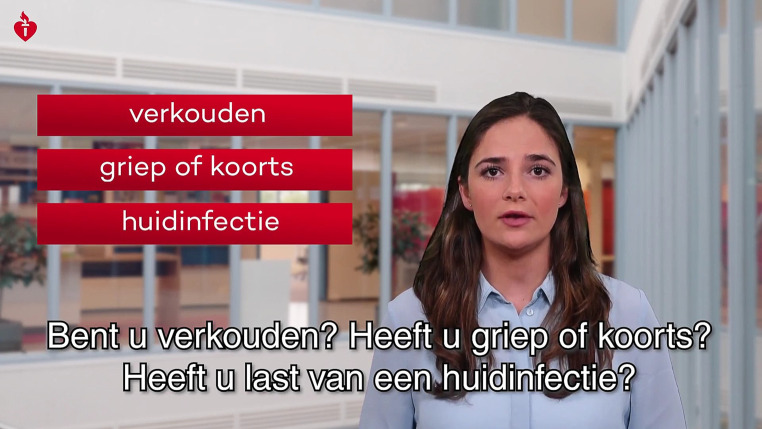
Still from the video ‘How to prepare for coronary artery bypass surgery?’

The aim of the videos is to prepare patients, and their caregivers and family members for the surgery and offer guidance during the recovery process. The videos were developed by the Dutch Heart Foundation and were made available specifically for this study. Videos contain spoken text with animations (Figs. 3 and 4); all information is in Dutch. A nurse practitioner experienced in care for cardiothoracic surgery patients will conduct the video consultations with patients on their recovery and any complaints. Supervision will be provided by a cardiothoracic surgeon. The nurse practitioner will be told the study’s aim is to ‘improve the current follow-up procedure’ and that he or she will therefore be blinded for the specific outcomes.

### Outcomes

#### Primary outcome measure

The primary outcome is the volume of unplanned healthcare utilisation (resources used per patient). Healthcare utilisation is defined as a composite endpoint of all emergency department visits, outpatient clinic visits, rehospitalisation, patient-initiated telephone consultations with a physician or nurse, and visits to a general practitioner, allied health professionals and psychologists.

An adaptation of the Institute for Medical Technology Assessment Medical Consumption Questionnaire (iMCQ) will be used to determine the volume of healthcare utilisation and the reasons thereof. This generic questionnaire aims to determine the costs of healthcare based on care consumption and is applicable to the Dutch healthcare system [10]. The questionnaire answers will result in absolute frequencies of visits for the relevant care activities. When patients report the use of healthcare, their healthcare provider will be contacted to validate the date and the reason for the healthcare encounter. This information will be crossvalidated with the subjects’ self-reports.

#### Secondary outcome measures

Secondary outcome measure are defined as the individual care activities of unplanned healthcare utilisation, a composite endpoint of consultations with a general practitioner, allied health professionals and psychologists plus patient-initiated telephone consultations with a physician or nurse, and a composite endpoint of in-hospital care (emergency department visits, outpatient clinic visits, rehospitalisation).

#### Tertiary outcome measures

Tertiary outcome measures are the patients’ quality of life and their mental and physical status. To assess these domains, the 36-Item Short Form Health Survey (SF-36) [11], the anxiety subscale of the Hospital Anxiety and Depression Scale (HADS) [12] and an adaptation of the Recovery Index-10 (RI-10) [13] will be used at different time points during follow-up (Tab. 2).

**Table 2:** Schedule of enrolment, interventions and assessments

	Time point^a^							
	Enrolment	Surgery	Follow-up					
Variable	*T*_*0*_		*T*_*1*_	*T*_*2*_	*T*_*3*_	*T*_*4*_	*T*_*5*_	*T*_*6*_
**Enrolment**								
– Eligibility screening	X							
– Informed consent	X							
– Allocation	X							
**Interventions**								
*Intervention group*								
– Standard care	X	X	X	X	X	X	X	X
– Online educational videos	X		X	X	X	X	X	X
– Video consultation				X		X		
*Control group*								
– Standard care	X	X	X	X	X	X	X	X
**Assessments**								
*Primary and secondary outcome measures*								
– Healthcare utilisation (iMCQ)						X		
*Tertiary outcome measures*								
– Quality of life (SF-36)	X							X
– Anxiety (HADS subscale)	X		X	X	X	X		
– Recovery (RI-10)			X	X	X	X		
*Process measures*								
– Patient satisfaction (satisfaction questionnaire)							X	
– Use of intervention (internet usage log)	X	X	X	X	X	X	X	X
*Patient and procedural data*								
– Sociodemographic data (iMCQ)	X							
– Patient characteristics (patient files)	X							
– Follow-up data (patient files)						X		

*iMCQ* Institute for Medical Technology Assessment Medical Consumption Questionnaire, *SF-36* 36-Item Short Form Health Survey, *HADS* Hospital Anxiety and Depression Scale, *RI-10* Recovery Index-10

^a^T_0_: 1 month before surgery; T_1_: 1 week after surgery; T_2_: 2 weeks after surgery; T_3_: 3 weeks after surgery; T_4_: 6 weeks after surgery; T_5_: 2 months after surgery; T_6_: 6 months after surgery

The SF-36 is routinely used at our facility and has been validated in multiple patient populations, including cardiac surgery patients [14–18]. This questionnaire assesses health-related quality of life in the previous 4 weeks. The HADS questionnaire is widely validated and is most commonly used to assess depression or anxiety. The RI-10 is a short, Dutch-language, 10-item questionnaire measuring postoperative domains on a 5-point scale over the last 7 days. It has been validated in gynaecology patients, but the general nature of the questions suits our patient population [19].

#### Process measures

The performance of the eHealth strategy on several domains will be structurally evaluated using qualitative and quantitative measures [20, 21]. The process evaluation will assess participant attitude, eligibility, access, usage and engagement of the eHealth strategy (Tab. 3), in order to make recommendations on the development and subsequent implementation of eHealth strategies. Data will originate from the internet usage log of the portal and the patient satisfaction questionnaire.

**Table 3:** Process evaluation of eHealth strategy

Domain *method of collection*	Portal	Video consultation
Eligibility *logistic data*	Number of patients not eligible for inclusion due to technological limitations (e.g. no computer access, digital illiteracy)	
Access *logistic data*	Number of patients that received access to an account	Number of patients that were invited via email
Usage *internet usage log*	Number of patients that logged in during the study period	Number of patients that completed the planned video consultation
	Time points at which patients logged in	Number of patients that experienced technical errors and the reasons thereof (e.g. magic link not working, bad quality of video connection)
	Average session length	
Engagement *internet usage log*	Number of videos started and completed during each visit	Average session length
	Number of times each video was started and completed	
Participants attitude *questionnaire*	Assessment of the portal/software (e.g. accessibility, interface, navigation) and reasons for not using the portal/software (if applicable)	
	Evaluation of content (comprehensible, use of words, useful)	Number of patients that deem video consultation a meaningful addition to standard care
	Number of patients that would recommend the content as a source of information to family members or other patients	

#### Patient and procedural data

Patient characteristics (age, sex, comorbidities), procedural characteristics (in-hospital complications, length of stay, duration of surgery), follow-up data (mortality, reoperations, deep sternal wound infections, stroke, recurrent myocardial infarction within 30 days) and sociodemographic information will be collected and analysed to provide insight into our patient population and to adjust endpoints if necessary. Patient, procedural and follow-up data are routinely collected at our facility and are defined by the Netherlands Heart Registry [22]. Sociodemographic data are part of the iMCQ.

### Data collection

Data will be collected using paper questionnaires at the following time points: 1 month before surgery (T_0_), 1 week after surgery (T_1_), 2 weeks after surgery (T_2_), 3 weeks after surgery (T_3_), 6 weeks after surgery (T_4_), 2 months after surgery (T_5_) and 6 months after surgery (T_6_) (Tab. 2). If patients do not return two subsequent questionnaires, they will be contacted and kindly requested to fill in and return the next questionnaires. Video consultation will be scheduled for 1 and 3 weeks after surgery.

### Statistical considerations

#### Data analysis

Descriptive statistics will be used to summarise baseline characteristics of the study population. Healthcare utilisation will be expressed as mean ± standard deviation (resource use per patient) and absolute and relative frequencies (users per resource). Mann-Whitney U test and Fisher exact test will be used to compare the intervention and control group. Multivariate regression analysis will be performed to adjust the outcomes for confounding factors based on univariate analysis (*p* < 0.1), literature review and expert opinion.

The primary analysis will be an intention-to-treat analysis. In the secondary analysis, we will compare the control group with the intervention group that used the educational videos at least once and completed the video consultations (‘users only’). A *p*-value <0.05 will be considered statistically significant and all analyses will be performed using SPSS 25 (SPSS Inc., Chicago, IL, USA).

#### Sample size calculation

Studies on eHealth in CABG patients and the effect on healthcare utilisation are scarce and report users per resource [23]. Our primary objective is to reduce healthcare utilisation per patient. To our knowledge, in one study, healthcare utilisation per patient was estimated using the iMCQ at 0.88 ± 0.15.[24] Under the assumption of a small or medium effect of our eHealth strategy (d = 0.35), an α of 0.05 and a power of 0.80, a total sample size of 260 patients is required. The total study population is set at 280 patients (140 patients per arm) to account for loss to follow-up and nonadherence to the intervention.

## Expected results

The IMPROV-ED trial will be carried out to evaluate whether an eHealth initiative consisting of online education and video consultation can reduce healthcare utilisation by improving quality of life, decreasing anxiety and accelerating recovery within the first 6 weeks after discharge for CABG.

## Discussion

The IMPROV-ED trial is of clinical significance for several reasons. First, we will evaluate the influence of an eHealth strategy on healthcare utilisation, anxiety, quality of life and recovery. Positive results will yield a new postoperative protocol that will lead to better patient outcomes and reduced costs [25]. In addition, the process and patient satisfaction evaluation will show the readiness of CABG patients for structured eHealth initiatives and will evaluate the currently used content and mode of administration, given the broad applicability of eHealth in general and the multitude of devices available [26–28]. Second, the control arm of the trial will provide the first detailed insight into unplanned, transmural healthcare utilisation in the early postoperative period after CABG and will thereby show how to further improve post-CABG protocols, aside from eHealth, through multidisciplinary regional collaboration.

eHealth strategies in CABG patients have been successfully applied to guide secondary prevention [29], to improve recovery [30, 31], and to assess physical functioning and quality of life [32–34]. Although evidence on the effect of eHealth on healthcare utilisation in CABG patients is minimal, it is reasonable to expect a positive effect based on reduction of healthcare utilisation by eHealth strategies in other populations [8, 23, 35, 36]. According to post-CABG protocols, patients are expected to adopt new behaviours (e.g. relieve stress on the sternum, gradually increase in physical exercise, follow healthy diet) and to deal with the emotions and worries that go with cardiac surgery through self-management, and, thus, to take responsibility for their own recovery [6]. eHealth has shown to be a useful method for patients to enhance their self-management through better understanding of their disease, increased independence and improved acceptance to adhere to lifestyle advice [37]. The educational videos in our eHealth strategy facilitate self-management. By means of video consultation, the physician can guide and supervise the patient’s progress and maintain a good patient-physician relationship, which has been shown to enhance the patient’s self-management skills [37].

The message and content of the educational videos were designed in such a way that they provide health information for patients with low/inadequate health literacy (approximately 36.4% of the general population in the Netherlands [38]), without compromising health communication to patients with adequate health literacy. Meppelink et al. have assessed the features of health information (written vs spoken text vs animations vs illustrations) and concluded that spoken text combined with animation is the most effective way to communicate health information and that it suits both patients with low health literacy and those with adequate health literacy [39]. In addition, to prevent cognitive overload, it is advised to only offer information when it is applicable to the patient’s situation instead of presenting all the information at once, especially to not overburden low health literate patients [40]. We therefore decided to divide the information into the three main phases of CABG recovery (Fig. 2).

### Limitations

The addition of our eHealth strategy to the postoperative protocol might yield additional costs in comparison to standard care. We believe that these additional costs will be balanced by reduced healthcare utilisation and will therefore result in less total costs and better patient outcomes. Another potential limitation is that we will only include patients that have sufficient computer and digital literacy skills and have access to a computer or tablet, which might diminish generalisability of our study protocol. Moreover, as in most eHealth research, our trial is not fully blinded, which could lead to bias when patients report healthcare utilisation.
